# Nucleoporin TPR Affects C2C12 Myogenic Differentiation via Regulation of *Myh4* Expression

**DOI:** 10.3390/cells10061271

**Published:** 2021-05-21

**Authors:** Jana Uhlířová, Lenka Šebestová, Karel Fišer, Tomáš Sieger, Jindřiška Fišerová, Pavel Hozák

**Affiliations:** 1 Department of Biology of the Cell Nucleus, Institute of Molecular Genetics of the CAS, 142 20 Prague, Czech Republic; jana.uhlirova@img.cas.cz (J.U.); lenka.sebestova@img.cas.cz (L.Š.); 2 First Faculty of Medicine, Department of Cell Biology, Charles University, 121 08 Prague, Czech Republic; 3 Faculty of Science, Department of Cell Biology, Charles University, 128 00 Prague, Czech Republic; 4 CLIP-Childhood Leukaemia Investigation Prague, Department of Pediatric Hematology/Oncology, Second Faculty of Medicine of the Charles University and University Hospital Motol, 150 00 Prague, Czech Republic; karel.fiser@lfmotol.cuni.cz; 5 Department of Cybernetics, Faculty of Electrical Engineering, Czech Technical University in Prague, 121 35 Prague, Czech Republic; siegetom@fel.cvut.cz; 6 Microscopy Center—LM and EM, Institute of Molecular Genetics of the CAS, 142 20 Prague, Czech Republic

**Keywords:** nucleoporin, translocated promoter region, TPR, LSD1, myogenic differentiation, gene expression, *Myh4*, *Olfr*

## Abstract

The nuclear pore complex (NPC) has emerged as a hub for the transcriptional regulation of a subset of genes, and this type of regulation plays an important role during differentiation. Nucleoporin TPR forms the nuclear basket of the NPC and is crucial for the enrichment of open chromatin around NPCs. TPR has been implicated in the regulation of transcription; however, the role of TPR in gene expression and cell differentiation has not been described. Here we show that depletion of TPR results in an aberrant morphology of murine proliferating C2C12 myoblasts (MBs) and differentiated C2C12 myotubes (MTs). The ChIP-Seq data revealed that TPR binds to genes linked to muscle formation and function, such as myosin heavy chain (*Myh4*), myocyte enhancer factor 2C (*Mef2C*) and a majority of olfactory receptor (*Olfr*) genes. We further show that TPR, possibly via lysine-specific demethylase 1 (LSD1), promotes the expression of *Myh4* and *Olfr376*, but not *Mef2C*. This provides a novel insight into the mechanism of myogenesis; however, more evidence is needed to fully elucidate the mechanism by which TPR affects specific myogenic genes.

## 1. Introduction

Nucleoporins (NUPs) are proteins that form a nuclear pore complex (NPC) and thus enable specific transport between the cytoplasm and the nucleus. In the past decade, NUPs have been intensively studied for their role in gene expression. They support chromatin decondensation [1] and mediate promoter–enhancer looping [2]. Furthermore, NUPs target genes, super-enhancer sequences and transcriptional factors to the areas around NPCs [2,3,4,5], which are associated with open, transcriptionally active chromatin [6]. This regulation is particularly important during differentiation, a process characterized by vast changes in gene expression profiles [3,7,8,9].

Translocated promotor region (TPR) is a large nucleoporin of 267 kDa that forms a nuclear pore basket along with NUP153 and NUP50 [10,11]. TPR plays many roles in the cell nucleus, including regulation of the TREX-2 dependent mRNA export pathway [12,13] and scaffolding for enzymes such as ERK2 [14] and MYC [4]. Similarly to many transcription-regulating NUPs, TPR localizes to the nucleoplasm [15]; however, its nucleoplasmic role has not yet been described. Furthermore, TPR binds chromatin in vitro [16] and is crucial for the forming of heterochromatin exclusion zones in the vicinity of NPCs [6]. As these areas are important for transcriptional regulation, a question arises as to the role of TPR in this process and during differentiation.

The C2C12 murine cell line represents an established model for myogenic differentiation. Proliferating C2C12 myoblasts (MBs) differentiate into myotubes (MTs) upon reaching full confluency on a Petri dish. In early differentiation stages, C2C12 cells express the transcription factors (TFs) and common differentiation markers myoblast determination protein 1 (MYOD1) and myogenic factor 5 (MYF5), later expressing myogenin (MYOG) and myocyte enhancer factor C (MEF2C; [17]) and finally, various muscle-specific myosin heavy chain (MYH) proteins, especially MYH4, a MYH that is characteristic of fast-twitching muscle fibers (reviewed in [18]).

Lysine-specific demethylase 1 (LSD1) is an enzyme that removes mono- and di-methyl groups from lysine residues. Depending on other proteins in the complex, LSD1 can serve as a transcriptional co-repressor via demethylation of lysine 4 at histone 3 (H3K4me1/2, [19]) and lysine 20 at histone 4 (H4K20me1/2, [20]), or as a transcriptional co-activator via demethylation of lysine 9 (H3K9me1/2, [21]). Furthermore, LSD1 demethylates and affects the activity of various non-histone targets, including TFs (reviewed in [22]). The role of LSD1 in myogenesis has been shown repeatedly in vitro [23,24,25,26,27,28] and in vivo using mouse models [25,26]. LSD1 promotes myogenic differentiation by activating demethylation of MYOD1 and MEF2D [23,24] and by removal of the repressive histone mark H3K9me1/2 from the *Myod* enhancer [25]. LSD1 knock-out leads to decreased expression of *Myod* and *Myog*, as well as *Myf6* and heavy chain myosins typical of both fast- and slow-twitching muscles such as *Myh1*, *Myh2*, *Myh4* and *Myh7* [23,24,26]. In contrast to these data, another study showed that LSD1 did not affect the expression of *Myh4* but inhibited the expression of genes related to slow-twitching fibers, i.e., *Myh7*, via the removal of the active histone mark H3K4me1/2, and thus shifted myogenic differentiation towards fast-twitching fibers [27]. In mice, LSD1 promotes myogenesis [25,28] and represses the brown adipocyte program in satellite cells during muscle regeneration by directly up- and downregulating the respective TFs [28].

Here, we show that TPR loss affects the myogenic differentiation and gene expression of muscle-specific genes. We provide data indicating that TPR targets LSD1 to these genes and we hypothesize the possible consequences of such an interaction.

## 2. Materials and Methods

### 2.1. Cell Culture, Differentiation, Plasmid Transfection, esiRNA Transfection and shRNA Transformation

C2C12, mouse C3H muscle myoblast cells (ATCC CRL-1772), were routinely maintained at low confluency (bellow 75%) in high-glucose DMEM (Sigma-Aldrich, St. Louis, MO, USA) supplemented with 10% fetal bovine serum (Sigma-Aldrich, St. Louis, MO, USA) and no antibiotics at 37 °C in 5% CO_2_ humidified atmosphere. Differentiation was induced at confluence in DMEM with 2% horse serum, standardly for 96 h. Non-differentiated myoblasts at 70% confluency (referred to as “MB”) and 100% confluency (referred to as “MT0”), and myotubes differentiated for 1 day (referred to as “MT1”), 2 days (referred to as “MT2”; Figure S2) and 4 days (referred to as “MTs”; Figure S1a) were used for the experiments.

Transfections with siRNA were performed using Lipofectamine ^®^ RNAiMAX reagent (Invitrogen, Carlsbad, CA, USA) according to the manufacturer’s protocol, right after passaging of the cells. MISSION^®^ siRNA Universal Negative Control #1 (SIC001, referred to as “*siNC*”), MISSION esiRNAs targeting *Lsd1* (EMU058651 referred to as “*siLsd1*”), and *Tpr* (EMU050881, referred to as “*siTpr*”) were obtained from Sigma-Aldrich (St. Louis, MO, USA); siRNA targeting *Myh4* (s70260, referred to as “*siMyh4*”) was obtained from Thermo Fisher Scientific (Waltham, MA, USA). To increase the efficiency of *Tpr* and *Lsd1* depletion, cells were re-transfected two days after the first transfection.

Stable C2C12 cell lines were prepared via lentiviral knockdown using empty pLKO.1 vectors (Sigma-Aldrich, referred to as “sh0”) or pLKO.1 vectors expressing shRNAs targeting *Tpr* transcripts in two different regions, as well as non-targeting shRNA (shNC; Table 1).

Lentiviral particles were produced in HEK 293T cells. HEK 293T cells were plated on 15-cm cell culture flasks (TPP Techno Plastic Products AG, Trasadingen, Switzerlandand) to 30% confluence and 24 h later co-transfected with 22.5 µg of shRNA vector, 10 µg of pMD2.G and 17.5 µg of dR8.91 packaging plasmids using polyethyleneimine (23966, Polysciences, Hirschberg an der Bergstraße, Germany). The viral particles were collected 48 and 72 h after transfection and precipitated by 10% PEG 6000. C2C12 cells were transduced by all the collected lentiviral particles during re-plating to 10% confluence. The transduction medium was replaced with fresh culturing medium 16 h after infection. Cells were selected 48 h after transduction by adding puromycin at a final concentration of 4 µg/mL for 21 days. Then puromycin concentration was decreased to 2 µg/mL for further maintenance.

Western blotting and quantitative RT-PCR were performed to screen transduced cells for effective TPR depletion and the two lines with the strongest TPR depletion were used for further experiments. The expression of TPR was elevated in shNC cells compared to WT and both sh0 and shNC were not able to differentiate appropriately (the abnormalities, however, differed from those found in shTPR cells, Figure S1). Thus, we decided to use WT cells as a control. To confirm the crucial results, siRNA experiments were performed to reduce the risk of off-target effects.

### 2.2. Western Blots

For protein extracts, cells were scraped, resuspended in 2× Laemmli non-reducing buffer (66 mM Tris-HCl, 26% glycerol, 2% SDS) and sonicated 10 × 30 s. The BCA assay (Thermo Fisher Scientific, Waltham, MA, USA) was used to adjust the protein concentration to the same levels. Then, 2% β-mercaptoethanol + 0.01% bromophenol blue were added, samples were incubated for 5 min at 95 °C and finally DMSO was added to yield a final concentration of 10 μM.

For Western blot analysis, 15 μg (for the staining of abundant proteins smaller than 70 kDa) to 80 μg (for TPR staining) of protein was loaded onto SDS-PAGE gels and then transferred to Immobilon-FL membranes (Millipore-Sigma, Burlington, MA, USA). Membranes were blocked with PBS-0.05% Tween (PBS-T) + 2% BSA for 1 h and incubated with the primary antibody for 1 h at room temperature (RT) or overnight (ON) at 4 °C and washed three times in PBS-T. The secondary antibody was added for 1 h at RT and washed three times in PBS-T. Membranes were imaged with an Odyssey infrared imaging system (LI-COR Biosciences, Lincoln, NE, USA). Protein levels in each sample were normalized to the level of α-tubulin. In WT MB cells, the protein level was set as 1 and assessed as a proportional change in differentiated or TPR-depleted cells. Statistical evaluation was based on biological replicates (for the exact number of replicates, see figure legends), the data were log-transformed prior to statistical evaluation. Student’s one-sample *t*-test was used when comparing to WT MB (WT MB values were used for the normalization between replicates and thus always equaled 1); Welch’s *t*-test was used when comparing to WT MT.

### 2.3. Immunofluorescence

For immunofluorescence, MBs and MTs were cultured on slides that were pre-coated with poly-L-lysine and then laminin (Sigma-Aldrich, St. Louis, MO, USA) according to the manufacturer’s instructions. The cells were fixed with 3% PFA for 20 min; washed 3 × 10 min with PBS; permeabilized with 0.5% Triton X-100 for 5 min; washed 3 × 5 min with PBS; blocked in 2% BSA in PBS-T; incubated with primary antibody in 2% BSA in PBS for 1 h at 37 °C; washed three times in 0.5% Tween in PBS (PBS-T); incubated with secondary antibody in 2% BSA for an additional hour at RT and washed in PBS-T buffer. Finally, the cells were incubated with Hoechst for 5 min and mounted using Vectashield (Vector labs). Images were acquired with an inverted DMi8 microscope with a confocal-head Leica TCS SP8 (lasers: 405 nm diode laser, 50 mW, 488 nm solid-state laser, 20 mW, 552 nm solid-state laser, 20 mW, 638 nm solid-state laser, 30 mW; objectives: HC PL APO 63×/1.40 OIL CS2, FWD 0.14, CG 0.17; detectors: photomultiplier tube (PMT) and supersensitive hybrid detectors (HyD); confocal head: acousto-optical tunable filter (AOTF), low Incident angle, dichroic beam splitters, standard scanner (1–1800 Hz line frequency), maximum scanner resolution 8192 × 8192 pixels, hardware zoom 0.75×–48×; dichroic mirrors: 488/552/638 nm triple excitation dichroic 488/552 nm dual excitation dichroic Substrate RT 15/85; immersion liquid: Type F immersion liquid (Leica Microsystems); software: Las X) and a Leica DM6000 (Leica Microsystems, Wetzlar, Germany; light source: Leica EL6000 with an HXP 120W/45C Vis Hg; filter cubes: A, I3, N2, Y3; objective: HCX PL FL L 40×/0.6 CORR PH2 XT; FWD 3.3-1.9; CG 0-2; Camera: Leica DFC350 FX; software: Las X). For visualization purposes, images were further deconvolved using Huygens Professional software (algorithm CMLE, theoretical PSF).

### 2.4. Antibodies

Primary antibodies were as follows: anti-TPR mouse monoclonal (TPR-N, ab58344, Abcam, Cambridge, UK, 1:300), anti-TPR rabbit polyclonal (TPR-C, ab84516, Abcam, Cambridge, UK, 1:300), anti-NUP98 rat monoclonal IgG2c (ab50610, Abcam, Cambridge, UK, 1:300), anti-NPC proteins mouse monoclonal (MAB414, ab24609, Abcam, Cambridge, UK, 1:300), anti-NUP153 rat monoclonal IgG2a (ab81463, Abcam, Cambridge, UK, 1:300), anti-MYOG mouse monoclonal (sc-52903, Santa Cruz Biotechnology, Santa Cruz, CA, USA, 1:300), anti-MEF2C rabbit monoclonal (ab211493, Abcam, Cambridge, UK, 1:500), anti-MYH4 rabbit polyclonal (ABIN6263466, Aviva Systems Biology, San Diego, CA, USA), anti-pan-MYH mouse monoclonal (MF20, Novus Biologicals, Centennial, CO, USA), anti-MYF5 rabbit polyclonal (ab125301, Abcam, Cambridge, UK, 1:1000), anti-MYOD1 rabbit polyclonal (ab203383, Abcam, Cambridge, UK, 1:1000), anti-P21 mouse monoclonal (sc6246, Santa Cruz Biotechnology, Santa Cruz, CA, USA, 1:500), anti-P57 rabbit monoclonal (ab75974, Abcam, Cambridge, UK, 1:500), anti-LSD1 rabbit monoclonal (C69G12, Cell Signalling Technology, Danvers, MA, USA), anti-Histone 3 rabbit polyclonal (H0164, Merck KGaA, Darmstadt, Germany), anti-H3K9me2 rabbit monoclonal (ab32521, Abcam, Cambridge, UK, 1:500), anti-H3K4me2 rabbit monoclonal (ab32356, Abcam, Cambridge, UK, 1:500) and anti-tubulin α (N-terminal structural domain, TU-01, aa 65–79, 1:100) mouse monoclonal, kindly provided by Dr. Pavel Dráber (Institute of Molecular Genetics of the Czech Academy of Sciences, Prague, Czech Republic).

Secondary antibodies for immunofluorescence were: goat anti-rat IgG (H+L) antibody conjugated with Alexa Fluor 488 (A21434, Invitrogen, Carlsbad, CA, USA), goat anti-rat IgG (H+L) antibody conjugated with Alexa Fluor 647 (A21247, Invitrogen, Carlsbad, CA, USA), goat anti-mouse IgG (H+L) antibody conjugated with Alexa Fluor 488 (A21236, Invitrogen, Carlsbad, CA, USA), goat anti-mouse IgG (H+L) antibody conjugated with Alexa Fluor 555 (A21424, Invitrogen, Carlsbad, CA, USA) and goat anti-rabbit IgG (H+L) antibody conjugated with Alexa Fluor 555 (A21429, Invitrogen, Carlsbad, CA, USA). Secondary antibodies for Western blotting were: goat anti-mouse IRDye^®^ 800CW donkey anti-mouse IgG (926-32212, Licor, Lincoln, NE, USA) and IRDye^®^ 680RD goat anti-rabbit IgG (926-68071, Licor, Lincoln, NE, USA).

### 2.5. Image Analysis

We developed a software tool for analyzing the fluorescence intensity (FI) of TPR inside the nucleus called “nuclear circle analysis”. Images were acquired with a Leica SP8 confocal microscope (see above). Nuclei were segmented according to DAPI staining overlaid with staining of central NUPs, namely Mab414 (recognizing the conserved domain FXFG repeats in NUPs, such as NUP62, NUP152 or NUP90) or anti-NUP98. The FI of TPR was collected in Matlab software (Release 2015a, The MathWorks, Inc., Natick, MA, USA) pixel by pixel (pixel size being 90 nm) along curves parallel to the nuclear periphery (NP) with decreasing perimeter, starting at the NP (the layer of pixels contouring the segmented nuclei) towards the nuclear center. We calculated mean FI from all pixels present within a 1.5-μm distance from the NP. The respective values represented the FI of TPR at the NP. The mean FI of remaining pixels towards the nuclear center was calculated and presented as the FI of TPR in the nucleoplasm. Background intensity was calculated based on the mean background FI (measured from areas with no cells) in all images separately for TPR-C and TPR-N antibodies and subtracted from measured NP and nucleoplasm FI values for the calculation of differences between data. The paired *t*-test was used to compare FI between NP and nucleoplasm; Welch’s *t*-test was used to compare mean FI values between cell lines (for the exact number of replicates see figure legends) within each experiment.

NPC density was measured using a macro in Fiji (ImageJ) in peripheral z-sections of Mab414 immunofluorescence images acquired with a Leica SP8 confocal microscope. Briefly, NPCs were thresholded, segmented and the information about all segmented objects (NPCs) was collected by the Particle Analyzer in Fiji (ImageJ). The ratio of object number and area was calculated to determine the NPC number/μm^2^. For statistical evaluation, Welch’s *t*-test was used to compare NPC density between MBs and MTs (for the exact number of replicates, see figure legends) within each experiment.

The MT width and fusion index was measured manually in Fiji (ImageJ) based on the images of phase contrast overlaid with DAPI and myogenin staining, acquired with a Leica DM6000 microscope. For MT width measurement, a line perpendicular to the MT fiber was drawn in the widest part of each MT. The length of each line was collected as MT width for at least 100 MTs in each sample. For statistical evaluation, Welch’s *t*-test was used to compare the square root transformed MT width in cells (for the exact number of cells, see figure legends) between cell lines within each experiment. The MT fusion index was calculated as the ratio of the number of nuclei in single MTs versus the total number of cells. Welch’s *t*-test was used to compare square root transformed MT width/fusion index data for cells (for the exact number of cells, see figure legends) between cell lines within each experiment.

### 2.6. Chromatin Immunoprecipitation

ChIP-grade protein A/G magnetic beads (26162, Thermo Fisher Scientific, Waltham, MA, USA) were pre-blocked with 3% BSA for 3 h at 4 °C and then incubated with 3 μg of respective antibody in PBS buffer containing 0.05% Tween and 2% BSA at 4 °C for 1 h. Beads were washed 3 times with 1× SDS buffer and kept on ice.

Approximately 15 × 10^6^ C2C12 cells/IP sample were grown on 15-cm^2^ dishes and cross-linked via the addition of formaldehyde (to 1% final concentration) to the attached cells. Cross-linking was allowed to proceed at room temperature for 10 min and was terminated with glycine (final concentration 0.125 M). Cells were washed with PBS and scraped into PBS containing 1 µM ABSF.

Cells were collected by centrifugation, resuspended in MNAse buffer (10 mM HEPES, 60 mM KCl, 15 mM NaCl, 0.32 mM sucrose, 4 mM CaCl_2_, 2× complete protease inhibitors (1 um AEBSF, 1 mm benzamidine, 50 μg/mL TLCK, 50 μg/mL TPCK, 10 μg/mL aprotinin, 1 μg/mL leupeptin, 1 μg/mL pepstatin A)) and sonicated 5 × 30 s, 5 µ, for MBs and 10 × 30 s, 5 µ, for MTs. Samples were incubated 5 min at 37 °C, then 0.24 μL/mL MNAse for MBs or 0.84 μL/mL MNAse for MTs and 1 μL/mL RNAse A were added, and samples were incubated at 37 °C for another 10 min. MNAse digestion was terminated by adding 2× SDS buffer (90 mM HEPES, 220 mM NaCl, 20 mM EDTA, 1% NP-40, 0.2% DeoxNa, 0.2% SDS) and samples were further sonicated 5 × 30 min. Lysates were centrifuged at 16,000× *g* for 15 min. DNA concentration was measured using a Qubit ds broad range kit (Q32850, Life Technologies, Carlsbad, CA, USA). The concentration of DNA in samples was adjusted to 25 μg/mL. Two milliliters of each sample were loaded onto beads and incubated overnight at 4 °C.

ChIP-Seq and ChIP-qPCR experiments were performed with the following combination of samples (Table 2 and Table 3, left) and antibodies (Table 2 and Table 3, top).

Immunoprecipitates were washed five times with 1× SDS buffer. Beads were resuspended in 200 μL of reverse-crosslink buffer (1% SDS, 100 mM NaHCO_3_, protein kinase K) and incubated at 55 °C for 1 h and then at 65 °C for 4 h. DNA was precipitated using isopropanol with the addition of glycogen, and washed 3× in ethanol. Pellets were resuspended in 40 μL of H_2_O and sent for sequencing or assayed by means of quantitative PCR.

### 2.7. Evaluation of ChIP-Seq Data

The fragmentation and quality of immunoprecipited DNA was analyzed using High Sensitivity DNA electrophoresis (Agilent, Santa Clara, CA, USA); the average fragment length was estimated as 300 bp. The preparation of DNA libraries and sequencing was performed at the EMBL Genomics Core Facility. ChIP-Seq primary single-end data were aligned by bowtie2 [29] against the mouse reference genome GRCm38 (mm10). Sequencing read quality and mapping quality was assessed using FastQC ([30], available online at: http://www.bioinformatics.babraham.ac.uk/projects/fastqc [15 February 2017] and qualimap [31] respectively. The aligned reads were further processed using MACS2 [32] with the settings: fragment size approximately 300 bp; sequenced reads from input samples served as a control.

We calculated the number of processed aligned reads within each gene range (reads/gene). For each gene we divided read/gene by the gene length to obtain the average number of reads per 1000 base pairs (reads/kbp). A threshold of at least 17 reads/kbp and 100 reads/gene was used to distinguish genes bound by TPR in MBs and MTs. We tested the thresholding at 6 genes via ChIP-qPCR. Indeed, the 3 genes (*Myh4*, *Olfr37*, *Mef2C*) with more than 17 reads/kbp in ChIP-Seq exhibited TPR binding above the threshold in ChIP-qPCR, in contrast to the three genes (*Myog*, *Myh7*, *Myod1*—the data for *Myh7* and *Myod1* are not shown) with less than 17 reads/kbp.

DESEQ2 [33] was used to analyze sample clustering and to calculate the difference in TPR binding assessed in MBs and MTs based on reads/gene. The sample clustering was based on the Euclidean distance calculated from variance stabilized data; complete linkage was used for heatmap [34]. The samples precipitated by the TPR-N (in both replicates) and TPR-C antibodies clustered together in MBs, as well as in MTs. Thus, the results gained by the two different antibodies were reproducible and were further approached as replicates. To calculate the difference in TPR binding in MBs and MTs, size factors and the dispersion for each gene were estimated for non-transformed data and a generalized linear model of the negative binomial family was fitted. *p*-values were calculated by means of the Wald test. Calculated *p*-values were further adjusted using the Benjamini–Hochberg adjustment (Padj).

The ontology enrichment of genes bound by TPR was examined in Perseus software [35] with 1D enrichment analysis [36], using the KEGG pathway database [37,38] and the GO database [39], of biological process (BP, GO:0008150), of cellular component (CC, GO:0005575) and of molecular function (MF, GO:0003674).

### 2.8. Quantitative PCR

C2C12 total RNA was isolated via Trizol-chloroform extraction and cDNA were synthesized using oligo(dT)20 primers from the Super-Script III First-Strand Synthesis SuperMix (18080051, Thermo Fisher Scientific, Waltham, MA, USA) as recommended in the manufacturer’s protocol. DNA for ChIP-qPCR experiments was prepared as described above.

qPCR was performed using the SYBR Green I master mix (04887352001, Roche Diagnostics GmbH, Mannheim, Germany) and primers (Table 4 and Table 5) and measured using a LightCycler^®^ 480 Instrument II (Roche Diagnostics GmbH, Mannheim, Germany) according to the manufacturer’s protocol under these conditions: 10 min at 95 °C, followed by 45 cycles of 95 °C for 15 s, 60 °C for 30 s and 72 °C for 15 s. The reactions were performed in triplicates from at least three independent experiments.

Levels of mRNA were evaluated for data with CT ≤ 30, using the 2^−ΔΔCT^ method and normalized to GAPDH as a reference gene. In WT MBs, *Tpr*, *Myog*, *Mef2C*, *Myh4*, *Olfr376*, *MymK*, *MymX*, *Myf5*, *MyoD1*, *P21* and *p57* mRNA levels were set at 1 and assessed as a fold change in differentiated or TPR-depleted cells. Data based on biological replicates (for the exact number of replicates, see figure legends) were log-transformed prior to statistical evaluation: Student’s one sample *t*-test was used when comparing to WT MB; Welch’s *t*-test was used when comparing to WT MT.

In ChIP-qPCR experiments, the level of genes immunoprecipitated by H3 was set as 1 and was assessed as a fold change in the same gene immunoprecipitated by TPR or LSD1. H3 = 1 was then set as a threshold for TPR and LSD1 binding to the gene. Data based on biological replicates (for the exact number of replicates, see figure legends) were square root transformed prior to statistical evaluation using Welch’s *t*-test.

## 3. Results

### 3.1. A Nucleoplasmic Pool of TPR Is Present in C2C12 Myoblasts and Diminishes in C2C12 Myotubes

**Figure 1:**
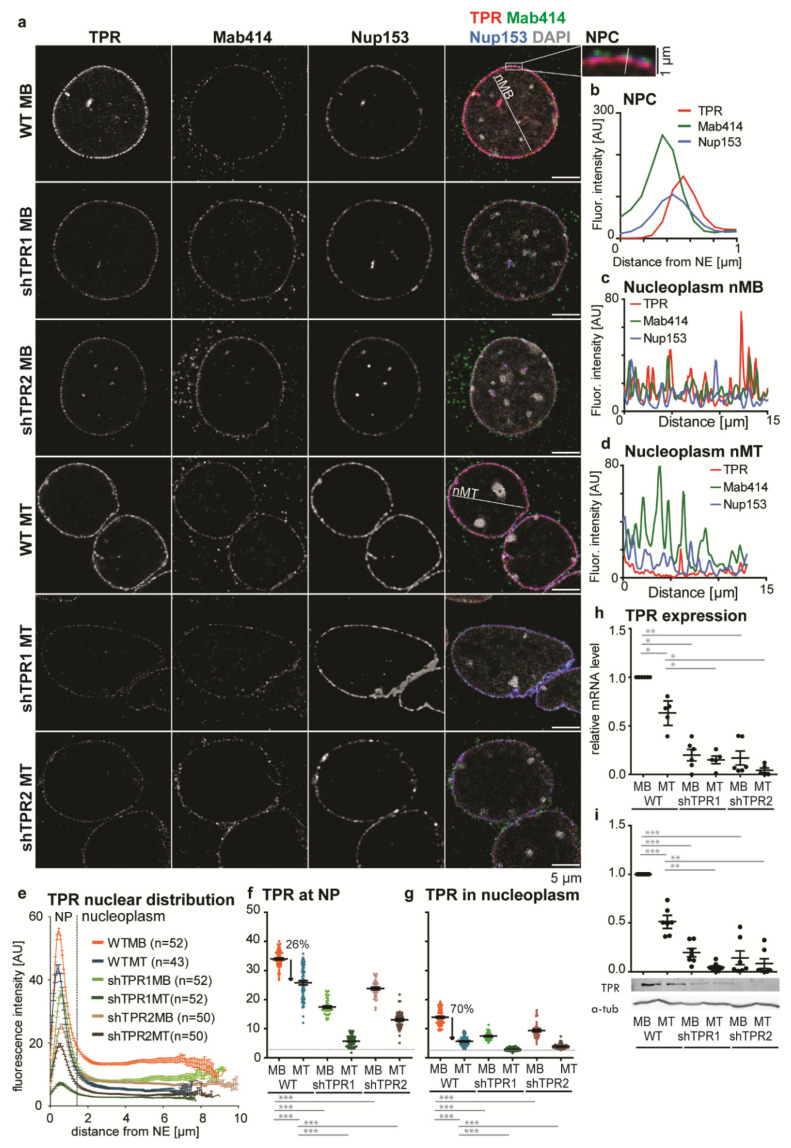
TPR localization and expression in the C2C12 cell line. (**a**) Nuclei of WT and TPR-depleted (shTPR1, shTPR2) C2C12 MBs and MTs (differentiated for 4 days) immunostained with anti-TPR-C (red, labeled with Alexa Fluor 555), Mab414 (staining central NUPs—green, labeled with Alexa Fluor 488) and NUP153 (blue, labeled with Alexa Fluor 647). Zoom-in shows spatial distribution of TPR, NUP153 and Mab414 within the NPC. (**b**) Plot profile depicts distribution of TPR, Mab414 and NUP153 at NPC. (**c**,**d**) Plot profiles show distribution of fluorescence intensities of TPR, NUP153 and Mab414 marked in Figure 1a in the nucleoplasm of WT MBs (nMB, C) and MTs (nMT, D). (**e**) Graph shows mean fluorescence intensities of TPR from the nuclear periphery (NP) towards the nuclear center in the mid z-stack of nuclei in WT and shTPR MBs and MTs. NP was considered an area of 1.5 µm from the nuclear periphery (segmented using central NUP staining) to the nuclear center. Data from one biological replicate out of three, n (WT MB) = 52, n (WT MT) = 43, n (shTPR1 MB) = 52, n (shTPR1 MT) = 52, n (shTPR2 MB) = 50, n (shTPR2 MT) = 50. (**f**,**g**) Dot plots depict means of TPR fluorescence intensity (FI) at NP and in nucleoplasm from Figure 1e. Statistics were evaluated for FI data subtracted from background FI values (gray dashed line). The paired *t*-test was used for nucleoplasm vs. NP comparisons; Welch’s *t*-test was used for between-sample comparisons. The decrease of TPR in the nucleoplasm upon differentiation was significantly higher than the decrease at NP: Welch’s *t*-test, *p* < 0.001. One dot represents one cell. (**h**) RT qPCR (*n* = 6) and (**i**) Western blotting quantification (*n* = 7) show the reduction in the TPR amount upon differentiation in WT MTs. qPCR and WB data were log-transformed prior to statistical evaluation: Student’s one-sample *t*-test was used to test against the WT MB values set to 1, Welch’s *t*-test was used for between-sample comparisons. One dot represents one biological replicate. Error bars represent s.e.m.; *, *p* < 0.05; **, *p* < 0.01; ***, *p* < 0.001.

Apart from their conventional role in nucleocytoplasmic transport, many NUPs also fulfil other roles in the nucleoplasm, i.e., they take part in transcriptional regulation [7,8,40]. Thus, we investigated precise nuclear TPR localization in undifferentiated C2C12 MBs and in MTs differentiated for four days (see Materials and Methods) by means of immunofluorescence, using two anti-TPR antibodies (TPR-N and TPR-C are targeted against the N- and C- terminus of TPR, respectively; see Materials and Methods). In MBs, TPR was located on the nucleoplasmic side of NPCs, as expected, but importantly also within the nucleoplasm (Figure 1a–c and Figure S1b). In MTs, TPR fluorescence intensity in the nucleoplasm was reduced (Figure 1a,d). Using our software tool (“nuclear circle analysis”; see Materials and Methods), we precisely analyzed the immunofluorescence data. Here, we present data acquired with the TPR-C antibody; the TPR-N antibody was used to confirm the trends (Figure S1c,d). We found that in MBs, the mean value of the TPR fluorescence intensity (FI) in the nucleoplasm reached ~40% of TPR intensity at the nuclear periphery (NP, Figure 1e–g). In MTs, the mean FI of TPR at NP decreased approx. four times (Figure 1f), in accordance with the reduction in NPC density in MTs (Figure S1e). The reduction in TPR mean FI was more prominent in the nucleoplasm of MTs and reached only 15% of FI at NP of MTs (Figure 1g and Figure S1d). RT-qPCR and a quantitative WB experiment confirmed that in MTs the TPR expression decreased to ~60% at the mRNA level (Figure 1h) and to ~50% at the protein level (Figure 1i).

Our data show that MBs contain a higher portion of nucleoplasmic TPR in comparison to MTs. Thus, functions of TPR that are associated with nucleoplasmic localization can occur in MBs more frequently.

### 3.2. TPR Affects C2C12 Differentiation

In order to study the role of TPR during C2C12 differentiation, we first aimed to prepare TPR knockout cells using CRISPR technology. The TPR knockout cells were not viable, however, and thus we prepared two C2C12 cell lines with TPR stably depleted by shRNAs (shTPR1, shTPR2). TPR expression in both shTPR1 and shTPR2 cell lines decreased to ~20% at the mRNA level (Figure 1h), as well as the protein level (Figure 1i), in both shTPR1 and shTPR2 C2C12 cell lines in MBs and MTs. We also inspected the level of TPR knockdown in shTPR cell lines using our software tool “nuclear circle analysis” in order to investigate if both compartments of TPR localization (NP and nucleoplasm) were affected to the same extent. In TPR-depleted MBs, the TPR FI reached only ~42% (shTPR1) or 65% (shTPR2) of FI at NP in WT MBs and ~43% (shTPR1) or 65% (shTPR2) of FI in the nucleoplasm in WT MBs (Figure 1a,e–g). The TPR depletion was more prominent in MTs, where the TPR FI reached ~10% (shTPR1) or 40% (shTPR2) at NP and only ~10% (shTPR1) or 30% (shTPR2) in nucleoplasm compared to WT MTs (Figure 1a,e–g). The overall nuclear pore density was, however, not affected by TPR depletion (Figure S1e), in accordance with the published data [41]. In conclusion, our data suggest that in TPR-depleted cells, the number of TPR molecules per NPC is reduced rather than total number of NPCs. Furthermore, the nucleoplasmic and NP pool of TPR are affected to the same extent.

**Figure 2:**
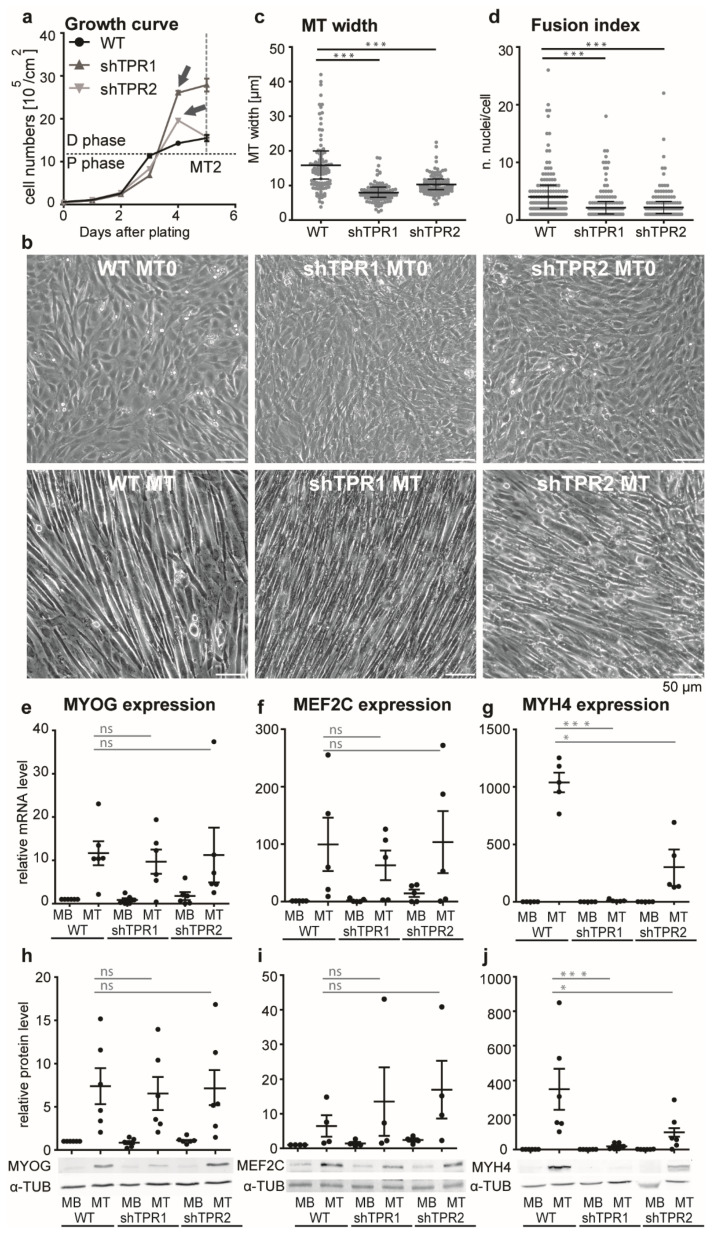
TPR is important for C2C12 myogenic differentiation. (**a**) Growth curve of WT and TPR-depleted C2C12 cells shows decreased proliferation rate (see proliferation phase, P) in TPR-depleted C2C12 cells. WT cells started to differentiate at a confluency of 10^6^/cm^2^. TPR-depleted cells continued to proliferate up to an almost tripled confluency compared to the WT (arrows, differentiation phase, D). The cells were counted until the second day of differentiation (gray dashed line, MT2. The graph represents one biological replicate. (**b**) Phase contrast images of WT and TPR-depleted fully confluent MT0 and MTs (differentiated for 0 and 4 days, respectively). (**c**,**d**) Dot plots show decreased MT width and fusion index in TPR-depleted C2C12 cells in comparison to WT. Each dot represents one biological replicate out of three. Data were square root transformed for statistical evaluation: Welch’s *t*-test, n (each cell line) = 100 cells, one dot represents one cell. (**e**–**g**) TPR depletion did not affect *Myog*, (n (WT, shTPR1) = 6, n (shTPR2) = 5) and *Mef2C* mRNA levels in MTs (n = 5), but resulted in a reduction of *Myh4* mRNA levels in MTs (n = 5). (**h**–**j**) No difference was observed for MYOG (n = 6) and MEF2C protein levels in MTs (n = 4); the decreased expression was confirmed for MYH4 protein levels in MTs (n = 6). Each dot represents one biological replicate. qPCR and WB data were log-transformed prior to statistical evaluation: Student’s one sample *t*-test was used to test against the normalized WT MB value set to 1; Welch’s *t*-test was used for between-sample comparisons. Error bars represent s.e.m.; *, *p* < 0.05; ***, *p* < 0.001.; ns, non-significant.

Next, we inspected the growth parameters of TPR depleted C2C12 cells. At first, the TPR depletion resulted in a decrease in the proliferation rate (Figure 2a, proliferation phase (P)). The decrease was significant on the 3^rd^ day after plating, according to Welch’s test, assessed based on three biological replicates. WT cells exited the cell cycle and initiated differentiation immediately after reaching a full confluency of about 10^6^ cells/cm^2^, and a reduction of fetal serum in culturing medium to 2%. TPR-depleted cells, however, continued to intensively proliferate for one more day, and exited the cell cycle two days later than WT. Thus, before TPR-depleted cells started to differentiate visually, they formed an array of about two to three times higher density (Figure 2a, differentiation phase (D), and Figure 2b). TPR-depleted MTs at the 4th day of differentiation were thinner compared to the WT MTs (Figure 2b,c) and their fusion index decreased (Figure 2d). In shTPR C2C12 cells, we examined the expression of early and late differentiation markers, *Myog*, *Mef2C* and *Myh4* (reviewed in [42]). The mRNA and protein levels of transcription factors *Myog* and *Mef2C* increased in differentiated cells, with no significant difference between WT and TPR-depleted cells (Figure 2e,f,h,i). We did not observe any changes in expression of these genes on the second day of differentiation either (Figure S2a,b), suggesting that in TPR-depleted cells the expression of these genes was triggered normally. On the other hand, the mRNA and protein levels of *Myh4* were reduced by approximately 50 times in TPR-depleted cells in comparison to WT MTs (Figure 2g,j). We also examined the expression of *Myog*, *Mef2C* and *Myh4* in C2C12 cells depleted of TPR using the siRNA approach (Figure S2c–n) and we confirmed the decreased expression of *Myh4* in the TPR-depleted cells. Importantly, depletion of MYH4 specifically by siRNA (Figure S3a) resulted in decreased MT width (Figure S3b,c), a phenotype similar to the TPR-depleted MTs (Figure 2b,c). These data suggest that the phenotype of TPR-depleted MTs is at least partially linked to the deregulated *Myh4* expression in these cells. Because of the affected fusion index in TPR-depleted cells, we also examined the expression of two proteins responsible for the myoblast fusion; myomaker (*MymK*) and myomixer (*MymX*, rewieved in [43]). Indeed, we found that TPR depletion resulted in decreased expression of both *MymK* and *MymX* on the first day of differentiation (Figure S3d,e).

To decipher whether the deregulation of the differentiation of TPR-depleted cells is linked to altered expression of earlier differentiation markers, we examined the expression of *Myf5* and *MyoD1*; however, we did not observe any difference between the control and TPR-depleted cells (Figure S3f–k). Lastly, we tested whether the failure of the TPR-depleted C2C12 cells to switch off proliferation under the differentiation stimuli could be explained by decreased P21 and P57 levels, as was shown in mouse muscles [44]. However, the protein levels of both P21 and P57 were elevated in TPR-depleted C2C12 MBs (Figure S3m,n,p,q) when compared to siNC MBs and did not significantly differ from the control once the differentiation was initiated (Figure S3l–q).

Our data show that TPR depletion leads to prolonged proliferation under differentiation stimuli, as well as to aberrations in the differentiation process. On the gene expression level, TPR depletion affected the regulation of *Myh4*, *MymK* and *MymX* expression. Although the *MymK* and *MymX* mRNA levels in TPR-depleted cells regained the levels of control cells on the fourth day of differentiation, the cellular thickness, fusion index and MYH4 levels in TPR-depleted MTs were not restored even after a prolonged differentiation time of 8 days (data not shown). This suggests that the complex phenotype of TPR-depleted cells was not caused by the delayed differentiation. Rather, it indicates that TPR directly affects the differentiation process in two crucial steps—first, when the cell exits the cell cycle at full confluency, and next, when the cells fuse and form the multinucleated MTs.

### 3.3. TPR Binds Megadomains of DNA That Partly Overlap with LADs

In order to examine the role of TPR in the regulation of expression during myogenesis, we performed ChIP-Seq experiments in C2C12 MBs and MTs. We immunoprecipitated TPR using TPR-N and TPR-C antibodies. Histone H3 trimethylated at lysine 36 (H3K36me3) served as a positive control. We added a second biological replicate using TPR-N (TPR-N2).

**Figure 3:**
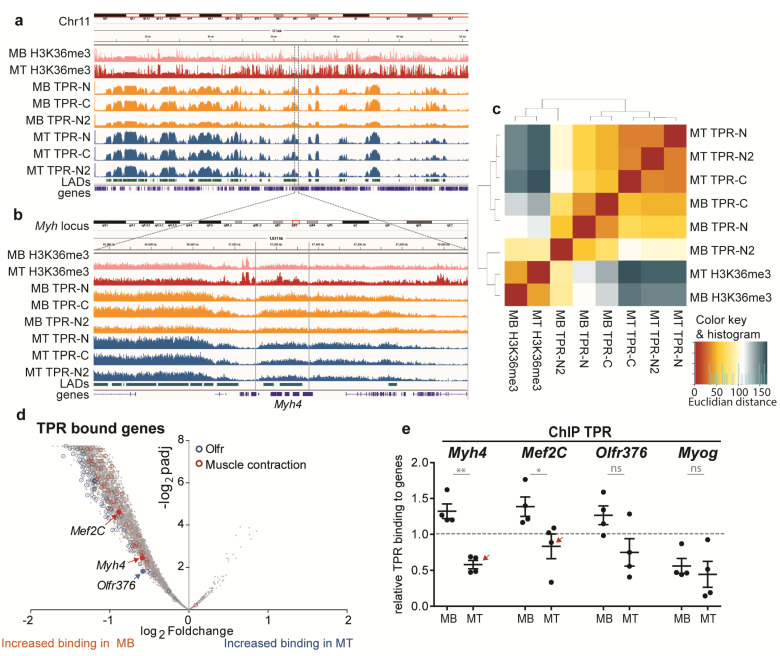
ChIP-sequencing reveals TPR binding in C2C12 MBs and MTs (differentiated for 4 days). (**a**) A comparison of binding patterns of H3K36me3 (top), TPR (middle) and Lamin B1-associated domains (LADs; bottom, [46]) within chromosome 11 in MBs and MTs. (**b**) Zoom-in to the TPR bound domain shows differential binding at *Myh4* between MBs and MTs (region of differential binding highlighted by gray lines). (**c**) Hierarchical clustering analysis of TPR binding in genes. Hits gained by TPR-N and TPR-C clustered together in MBs and MTs. (**d**) Volcano plot reveals the distribution of genes depending on *p*-value, adjusted for multiple correction (Benjamini–Hochberg adjustment, padj), and fold change of TPR binding between C2C12 MBs and MTs. Olfactory receptor genes are marked in blue and genes related to muscle contraction in red. Highlighted *Mef2C*, *Myh4* and *Olfr376* are representative genes for further experiments. (**e**) ChIP–qPCR confirms the ChIP-Seq data (n = 5): TPR binds *Mef2C*, *Myh4* and with decreased values also *Olfr376* in MBs; the binding is reduced in MTs. TPR binding to *Myog* was below the threshold. Data normalized to H3 binding in respective samples: H3 binding is equal to 1 = threshold. qPCR and WB data were square root transformed prior to statistical evaluation: Welch’s *t*-test. Each dot represents one biological replicate; error bars represent s.e.m. *, *p* < 0.05; **, *p* < 0.01; ns, non-significant.

Our ChIP-sequencing data of H3K36me3 in MBs and MTs corresponded with the previously published data [45] and revealed peaks present mostly within the gene bodies, e.g., in the *Myh* locus (Figure S4a). In contrast to H3K36me3, TPR bound chromatin in regions of mixed sizes. One type of bound regions was large, up to 5-Mbp-long portions of the genome (Figure 3a). These domains covered ~30% of the mouse genome and overlapped with Lamin B1-associated domains (LADs), as reported by Wu and Yao [46]. The overall position of these large, TPR-associated domains did not change in MTs. However, inside of the large TPR-associated regions, there were much smaller regions of pronounced TPR binding, which changed upon differentiation (Figure 3b, region between gray lines).

### 3.4. TPR Is Present at Myh4 and Other Genes Associated with Muscle Differentiation and Promotes Their Expression

In MBs, about 30% of TPR binding was found in genes. Hierarchical clustering analysis of TPR binding within genes revealed that the samples precipitated by the TPR-N (in both replicates) and TPR-C antibodies clustered together in MBs, as well as in MTs (Figure 3c). Using the Deseq2 tool, we identified changes in TPR binding in genes during differentiation and found a large portion of genes with stronger TPR binding in MBs, but only few with increased TPR binding in MTs (Figure 3d). Ontology enrichment analysis revealed gene groups with a high percentage of genes bound by TPR. These groups were often linked to myogenic differentiation and muscle cell functioning (Figure 3d and Figure S4b–d). Importantly, we observed TPR binding at *Myh4* and *Mef2C* (Figure 3d). An interesting hit was also presented by olfactory receptor genes (*Olfrs*), as these receptors have emerged as important factors for the differentiation of various tissues, including muscles [47,48]. We chose *Myh4*, *Mef2C* and *Olfr376* (Figure 3d) as representative candidates for further experiments. As a negative control for the ChIP-Seq data, we used *Myog*, which was neither bound by TPR, nor in MBs, nor in MTs. In accordance with this, the ChIP-qPCR confirmed that TPR binding to *Myh4*, *Olfr376* and *Mef2C* was above the threshold in MBs, in contrary to binding to *Myog* (Figure 3e). Furthermore, TPR binding to *Myh4* and *Mef2C* significantly decreased upon differentiation (Figure 3e, arrows), in accordance with the trend observed in the ChIP-Seq data (Figure 3d). The binding to *Olfr376* exhibited a similar trend, although the result was not significant.

Interestingly, in the shTPR C2C12 cell lines, we observed a decrease in the mRNA as well as protein levels of *Myh4* especially in MTs (Figure 2g,j) and *Olfr376* in both MBs and MTs (Figure S4e). On the other hand, the expression of *Mef2C* was unaffected by the TPR depletion (Figure 2f,i). The expression of *Myog,* which is not bound by TPR (Figure 3e), showed no correlation with TPR depletion (Figure 2e,h).

In conclusion, our data suggest that TPR affects the expression of TPR-bound genes *Olfr376* and *Myh4*, but not *Mef2C*. This might implicate different downstream regulation of *Mef2c* in comparison to *Olfr376* and *Myh4*.

### 3.5. TPR Targets LSD1 to Myh4 and Olfr376 but Not to Mef2C

**Figure 4:**
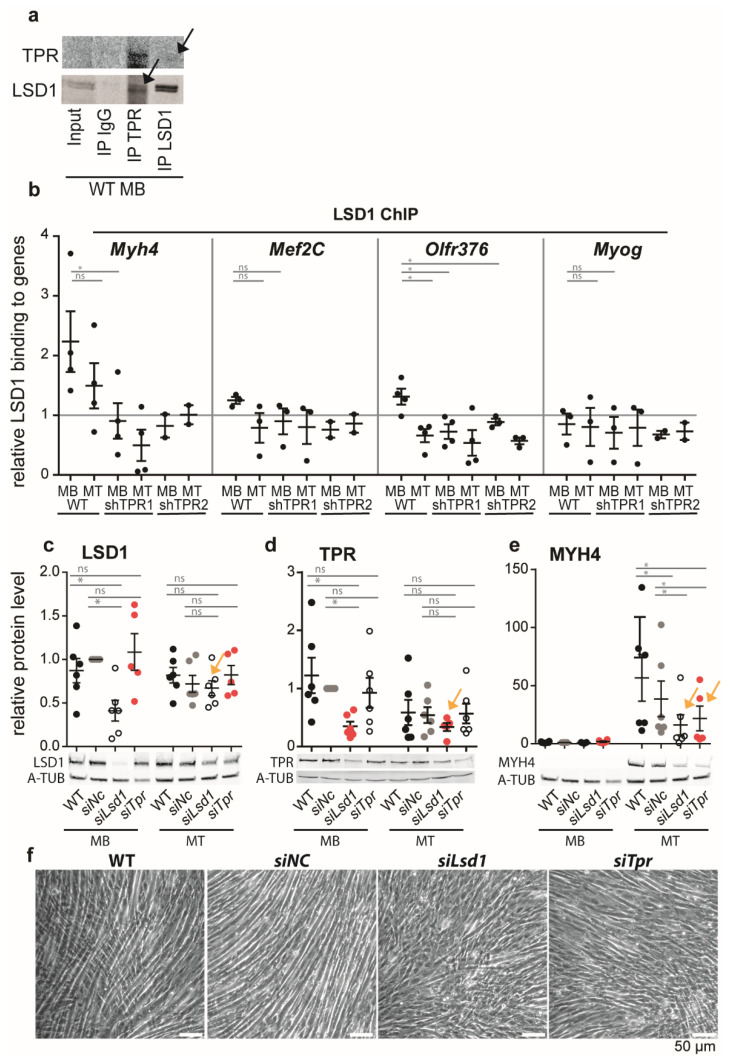
TPR co-immunoprecipitates with LSD1 in C2C12 MBs and targets it to *Myh4*. (**a**) Immunoprecipitation of TPR by anti-LSD1 (top), immunoprecipitation of LSD1 by anti-TPR-C (bottom) in C2C12 MBs. IgG served as a negative control. (**b**) ChIP-qPCR analysis of LSD1 binding to *Myh4* n (WT, shTPR1) = 4, n (shTPR2) = 2; *Mef2C* n (WT, shTPR1) = 3, n (shTPR2) = 2, *Olfr376* n (WT, shTPR1) = 4, (shTPR2) = 3; and *Myog* n (WT, shTPR1) = 3, n (shTPR2) = 2. Data normalized to H3 binding in respective sample; H3 binding is equal to 1 = threshold. Data were square root transformed prior to statistical evaluation using Welch’s *t*-test. Each dot represents one biological replicate. (**c**–**e**) Protein levels of LSD1, TPR and MYH4 in C2C12 control (WT, or transfected by non-targeting siRNA; siNC) and C2C12 cells depleted of LSD1 (siLsd1) or TPR (siTpr) using esiRNAs. Each dot represents one biological replicate, n (WT, siNC, siLSD1) = 6, n (siTPR) = 5. (**f**) Phase contrast images of MTs (differentiated for 4 days) arising from WT, siNC, siLsd1 and siTpr depleted MBs. Error bars represent s.e.m.; *p* < 0.01; *, *p* < 0.05; ns, non-significant.

To examine a possible mechanism by which TPR affects the expression of *Myh4* and *Olfr376*, we focused on histone-modifying enzymes. We reasoned that TPR may form a scaffold for such enzymes, as reported previously [4,14], and via changes in the histone modification pattern it may affect the epigenetic code of the TPR-bound genes. Our preliminary data suggested that TPR co-immunoprecipitated with LSD1 histone demethylase in HeLa cells (Fišerová, unpublished). To study LSD1 as a possible cofactor of TPR in muscle differentiation, we first confirmed that TPR was associated with LSD1 in MBs (Figure 4a). Quantitative WB experiments revealed that LSD1 expression decreased in MTs but was not affected by TPR depletion (Figure S5a). Accordingly, TPR depletion did not lead to significant changes in the overall levels of well-known histone marks regulated by LSD1, H3K9me2 and H3K4me2 (Figure S5b,c), although we observed a trend of a moderate decrease in H3K9me2 in WT MTs but not in TPR-depleted MTs (Figure S5b).

We hypothesized that TPR could target LSD1 to activate the promoters of genes. We tested this hypothesis using ChIP-qPCR in TPR-depleted MBs and MTs. In WT cells, LSD1 binding to *Myh4* decreased in MTs (Figure 4b). In the TPR-depleted MBs and MTs, LSD1 binding to *Myh4* decreased below the level of the threshold (Figure 4b). We observed the same trend for the *Olfr376* gene (Figure 4b). LSD1 binding to *Myog* was below the threshold in WT, as well as in TPR-depleted C2C12 cells (Figure 4b). Interestingly, LSD1 binding at the *Mef2C* gene was also not significantly affected by TPR depletion (Figure 4b). This result correlated with the above results showing that the expression of *Mef2C* was not affected by TPR depletion (Figure 2f,i) in contrast to *Myh4* and *Olfr376*.

The binding of TPR and LSD1 to *Myh4* was more prominent in MBs (Figure 3e and Figure 4b), although *Myh4* expression in MBs is minimal (Figure 2g,j). We hypothesized that the presence of TPR might, together with LSD1, affect the poised state of the gene, and prepare it for rapid expression as soon as the differentiation starts. To test this hypothesis, we depleted TPR or LSD1 in C2C12 MBs using siRNAs and allowed the cells to differentiate (Figure 4c,d). The resulting MTs exhibited decreased MYH4 expression, up to ~50% (Figure 4e, arrows), and altered MT morphology (Figure 4f), although the depletion of TPR and LSD1 were lost at the stage of MTs (Figure 4c,d, arrows). In other words, the absence of LSD1in MBs significantly affected the expression of the TPR-bound gene MYH4 in MTs, which suggests a preceding action of LSD1.

LSD1 can activate bound genes via the removal of the repressive H3K9me2 histone mark, often when in a complex with the androgen receptor (AR, [21]). Thus, we tested if TPR depletions result in increased H3K9me2 deposition at *Myh4* and *Olfr376* genes in shTPR MTs.

However, TPR depletion did not result in an increased abundance of H3K9me2 at *Myh4* and *Olfr376* (Figure S5d), nor did we find TPR in a complex with AR (data not shown).

To conclude, our data suggest that the binding of LSD1 to *Myh4* and *Olfr376* during the differentiation of C2C12 promotes the expression of both genes and is TPR-dependent. Below, we discuss the possible mechanism of LSD1′s action on the TPR-bound genes.

## 4. Discussion

In this study, we show that TPR is essential for the proper myogenic differentiation of C2C12 cells. It binds to the genes associated with muscle differentiation (the binding is often increased in non-differentiated MBs) and promotes the expression of at least some of the associated genes. Finally, TPR forms a complex with histone demethylase LSD1 that has been previously implicated in muscle gene regulation [23,24,25,26,27,28], and targets it to the TPR-associated genes.

Interestingly, we observed the altered phenotype even when TPR was depleted only transiently to 50% of control protein levels. Given the fact that we were not able to prepare a stable TPR-knockout cell line, we speculate that TPR is essential for the viability of cells and even a relatively mild decrease in protein levels has a visible impact. TPR depletion results in a decreased proliferation rate of C2C12 MBs, which is correlated with the elevated protein levels of P21 and P57. A similar phenotype was described in TPR-depleted HeLa cells [49,50], where the decreased proliferation rate was linked to cellular senescence [50]. Furthermore, C2C12 MBs show prolonged proliferation under differentiation stimuli, e.g., close cell-to-cell contact. Interestingly, the prolonged proliferation overlaps with the differentiation onset, which is marked by the elevated expression of genes triggering early stages of differentiation (such as MYOG and MEF2C). This means that the differentiation process has been initiated on the gene expression level in the TPR-depleted cells. Nevertheless, the differentiation was abrogated, as cell cycling continued and nuclear movement and fusion had not been initiated. We tested if the failure of the TPR-depleted C2C12 cells to switch off proliferation under the differentiation stimuli could have been explained by decreased P21 and P57 levels, as was shown for mouse muscles [44]. However, our data did not confirm this hypothesis, as the protein levels of both P21 and P57 in TPR-depleted cells did not significantly differ from the control once the differentiation was initiated (Figure S3l–q). Thus, we assume that other factors stimulating cell cycling are involved.

Furthermore, TPR depletion affects the development of muscle cells, as TPR-depleted C2C12 MTs are thinner and have a lower fusion index. We presume that TPR depletion would alter the development of muscle tissues at the organismal level as well. Indeed, TPR-knockout mice die before weaning (published at http://www.informatics.jax.org/allele/allgenoviews/MGI:5609352, accessed 15 December 2016, which could be the result of aberrant development of the tissues that become indispensable after birth), including heart muscle and smooth muscles in the lungs and gastro-intestinal system.

TPR localizes to the nucleoplasm of C2C12 MBs, similarly to many of the NUPs regulating gene expression [2,7,8,9,40]. The nucleoplasmic pool of TPR diminishes upon differentiation and TPR binding in a tested subset of TPR-associated genes decreases. Thus, we speculate that chromatin associates with TPR both at NPCs and in the nucleoplasm in MBs. In MTs, the nucleoplasmic TPR–chromatin association is decreased and the binding at NPC prevails. The chromatin associated with TPR consists of mega-base-pai-long domains, present mostly in gene-poor regions. This TPR pattern partly overlaps with lamina-associated domains (LADs, [46]). A similar binding pattern was described only for NUP153 binding in *D. melanogaster* [40]; other NUPs exhibited rather sharp peaks of binding to the chromatin, often present at the promoter regions and gene bodies [2,9,51]. The association of genes with the nuclear lamina (reviewed in [52]) or NUP153 [40] is often linked to transcriptional repression. NUP153 depletion results in the de-repression of developmental genes and the induction of early differentiation. On the contrary, TPR seems to have a positive effect on the expression of its associated genes, i.e., *Myh4* and *Olfr376*.

Our ChIP-Seq data revealed that many of the TPR-associated genes are responsible for muscle differentiation and functioning. Among these are *Myh4* and *Mef2C*, both of which are heavily expressed in C2C12 MTs (reviewed in [46]). We found that TPR affects the expression of *Myh4*. MYH4 represents a major muscle myosin in mice, and *Myh4*-knockout mice exhibit several abnormalities, such as decreased body weight, body strength and an altered myofibril phenotype [53]. Importantly, we found that the phenotype of MYH4-depleted MTs resembles that of TPR-depleted MTs. Taken together, we hypothesize that the phenotype of TPR depletion is linked, at least partially, to the aberrant expression of *Myh4*.

Interestingly, TPR binds to the majority of *Olfrs*. These receptors have been reported repeatedly to work also outside of the olfactory tissue, and importantly, are also implicated in the development of striated muscles and airway smooth muscles ([46,48], reviewed in [54]). In the striated muscles, only the function of OLFR16 has been described so far [48]. However, in other tissues, multiple OLFRs play a role in diverse functions; thus, it is unlikely that OLFR16 is the only one affecting muscles. Since TPR neither binds *Olfr16* nor affects its expression, we focused on *Olf376*, which was associated with TPR. Our data show that *Olfr376* is expressed in C2C12 MBs and its expression is positively regulated by TPR. We speculate that TPR might affect other *Olfrs* in a similar manner. The role of OLFRs in C2C12 cells, as well as the mechanisms of their expression regulation within the whole tissue and within individual nuclei of multinucleated sarcomere, remains an open question.

Both TPR binding to *Olfr376* and the expression of *Olfr376* are increased in MBs when compared to MTs. TPR depletion results in a decrease in *Olfr376* mRNA both in MBs and MTs. The positive correlation here clearly suggests a positive role of TPR in the expression of *Olfr376*. The situation is more complicated concerning *Myh4*. TPR binds to the *Myh4* gene in WT MBs when the *Myh4* expression is minimal. TPR binding decreases during differentiation, and the *Myh4* expression is rapidly elevated. Here, the negative correlation could implicate that TPR suppresses the expression of *Myh4*. However, TPR depletion leads to the decreased expression of *Myh4* in MTs. Furthermore, when we transiently depleted TPR in MBs, MYH4 expression was decreased in differentiated MTs 6 days post-transfection, even though the effect of TPR depletion was already lost. These data suggest that TPR may affect the poised state of the *Myh4* gene in MBs, rather than repressing the active gene in MTs.

Lastly, we showed that TPR depletion resulted in decreased expression of *MymK* and *MymX* in the early stage of differentiation. MYMK and MYMX are membrane proteins responsible for myoblast fusion (reviewed in [43]). Their lowered expression in TPR-depleted cells on the first differentiation day may have contributed to the lowered fusion index of the TPR-depleted MTs. In contrast to *Myh4*, *MymK* and *MymX* genes, which are not bound by TPR, their expression equaled the expression in control cells on the second day of differentiation. Because of this, we assume that the expression of *MymK* and *MymX* is not regulated directly by TPR. It is also unlikely that TPR would affect the expression of *MymK* and *MymX* by altering the expression of MYOD1 and MYOG, the two TFs described to promote the expression of the respective genes [55,56], as the TPR depletion did not affect the expression of either MYOD1 or MYOG. TPR affects an export of short, intron-less and intron-poor mRNAs [13] and consequently promotes the transcription of genes encoding for the respective mRNAs [12] via the TREX-2 pathway. *Myh4* mRNA contains multiple exons and thus is not the subject of TPR-dependent TREX-2 regulation. On the other hand, mRNAs of *MymK* and *MymX* possess three introns and one intron, respectively. Thus, in agreement with the published data, we hypothesize that TPR may regulate the expression of *MymK* and *MymX* by targeting the TREX-2 complex subunits to the nuclear pores.

To address the mechanism by which TPR promotes the expression of *Myh4* and *Olfr376*, we focused on histone-modifying enzyme LSD1. LSD1 is crucial for muscle formation [23,24,25,26,27,28] and its depletion leads to the decreased expression of several myogenic TFs and heavy chain myosins, including *Myh4* (Figure 4e, [23,26]). Moreover, LSD1-depleted MTs exhibit decreased MT width (Figure 4f,g) and fusion index [26], a phenotype similar to TPR-depleted MTs. We confirmed that TPR interacts with LSD1 in C2C12 MBs. Furthermore, we showed that LSD1 binds to *Myh4* and *Olfr376* genes in C2C12 MBs and that the LSD1 binding decreases upon differentiation or TPR depletion. Similarly to TPR, LSD1 binding to *Myh4* is negatively correlated with *Myh4* expression in WT cells. Furthermore, MYH4 expression in MTs was decreased 6 days after transient depletion of LSD1, when the LSD1 expression was already recovered. Furthermore, LSD1 poorly binds *Mef2C* (Figure 4b), the expression of which is not affected by TPR depletion. Together, these data suggest that LSD1 might be the factor co-regulating gene expression along with TPR. We speculate that in MBs, the TPR-LSD1 complex affects the poised state of the *Myh4* gene, preparing it for rapid expression once the differentiation process is initiated.

We hypothesized that LSD1 can activate the expression of genes via removal of the repressive H3K9me2 histone mark. LSD1 gains this transcriptionally promoting property when in a complex with AR [21]. However, we did not find TPR in a complex with AR, and nor did we detect the increased abundance of H3K9me2 at the *Myh4* or *Olfr376* locus in TPR-depleted MBs or MTs (Figure S5d). Thus, we hypothesize that TPR-targeted LSD1 might de-methylate TFs, such as MEF2D or MYOD1, at the *Myh4* and *Olfr376* promoters, as described by [24]. Another possibility is that LSD1 targets TFs to the TPR-associated genes, as shown for TF GATA2, which promotes RNA polymerase II recruitment and activates the transcription of genes crucial for the fusion of trophoblast cells into syncytiotrophoblasts [57]. Alternatively, the deregulation of *Myh4* and *Olfr376* in TPR-depleted cells could occur independently of LSD1, for instance through gene organization changes within the cell nucleus. 

Our results map the DNA-binding profile of the nucleoplasmic NUP TPR and show that TPR has a role in gene expression regulation during myogenesis. Furthermore, LSD1 was identified as a possible effector of TPR-regulated transcription of muscle genes. This provides a novel insight into the process of myogenesis; however, more evidence is needed to fully elucidate the mechanism by which TPR affects specific myogenic genes, and to find other factors involved in this process.

## Figures and Tables

**Table 1:** *Tpr* sequences targeted by the shRNA.

	Targeted mRNA	Targeting Sequence
shNC	None	TAAGGTTAAGTCGCCCTCGAT
shTPR1	TPR C-terminus, 5916–5936	TCTCCATCAGTACTTTCTTCT
shTPR2	TPR C-terminus, 5989–6009	TGTATAATCTCCCTGGGTAAC

**Table 2:** Combination of the samples and antibodies for ChIP-Seq.

ChIP-Seq	IgG	TPR-C	TPR-N	H3K36me3
C2C12 MB	X	X	X	X
C2C12 MT	X	X	X	X

**Table 3:** Combination of the samples and antibodies for ChIP-qPCR.

ChIP-qPCR	H3	TPR	LSD1	H3K9me2
C2C12 MB	X	X		
C2C12 MT	X	X		
shTPR1 MB	X	X	X	X
shTPR1 MT	X	X	X	X
shTPR2 MB	X	X	X	X
shTPR2 MT	X	X	X	X

**Table 4:** Primers used for ChIP-qPCR.

ChIP-qPCR	Forward	Reverse
*Mef2C*	CCAAAGTCCTCTCCTATGTGCTT	AGGCGCTTCACCTAACCAAG
*Myh4*	ACGCTGAGATGGCCGTTTTC	CTCCCACGTCTTGCTTTTACTC
*Olfr376*	TGAATGTGTGCTCCTCTCTATG	CACTTACTTACCACCACCATCAC
*Myog*	AGCCTTTTCCGACCTGATGG	CCCCATCATAGAAGTGGGGC

**Table 5:** Primers used for RT-qPCR.

RT-qPCR	Forward	Reverse
*GAPDH*	GGAAGGGCTCATGACCACAG	GCCATCCACAGTCTTCTGGG
*TPR*	TCCAGGCATATCAGAGCGA	CACCAGGCTGACCTTTACCG
*Mef2C*	AACATTTGCCAAAAGCGGCA	GTGACAGGCGTGTTCCTACA
*Myh4*	CACCCTGGAGGACCAACTGA	TTGCCTCGGGAAAGCTGAGAAA
*Olfr376*	ATATGAGCCAGATGCAGGGC	GCCAGGAACAGGGCATAGAA
*Myog*	GTCCCAACCCAGGAGATCATT	AGTTGGGCATGGTTTCGTCT
*MymK*	CGATTCTTCTTTGAGGAATGGGA	TCCCAGCCTTCTTGTTGACC
*MymX*	AGCAGGAGGGCAAGAAGTTCA	CTCATGTCTTGGGAGCTCAGT
*MyF5*	CGGATCACGTCTACAGAGCC	GCAGGAGTGATCATCGGGAG
*MyoD1*	TACAGTGGCGACTCAGATGC	GGCCGCTGTAATCCATCATGC
*P21*	CAGACCAGCCTGACAGATTTCTA	GAGGGCTAAGGCCGAAGATG
*P57*	GAAGTTGAAGTCCCAGCGGT	ACCAATCAGCCAGCAGAACAG

## Data Availability

Not applicable.

## Supplementary Materials

The following are available online at https://www.mdpi.com/article/10.3390/cells10061271/s1, Figure S1: TPR localizes at NPCs and in the nucleoplasm in C2C12, as confirmed by the TPR-N antibody. Figure S2: The effect of TPR depletion on the expression of *Myog*, *Mef2C* and *Myh4*, confirmed by the siRNA approach and in the earlier differentiation stages. Figure S3: The phenotype of TPR-depleted MTs is linked to the decreased expression of *Myh4*, *MymK* and *MymX*. Figure S4: ChIP-Seq analysis of TPR binding to the genome of C2C12 MBs and MTs. Figure S5: TPR does not affect total protein levels of LSD1, H3K9me2 in C2C12 MBs and MTs.
