# Supplementary figures and images for: Nucleoporin TPR Affects C2C12 Myogenic Differentiation via Regulation of Myh4 Expression
Source: Cells. 2021 May 21;10(6):1271. doi: 10.3390/cells10061271 (PMC8224082; doi:10.3390/cells10061271)

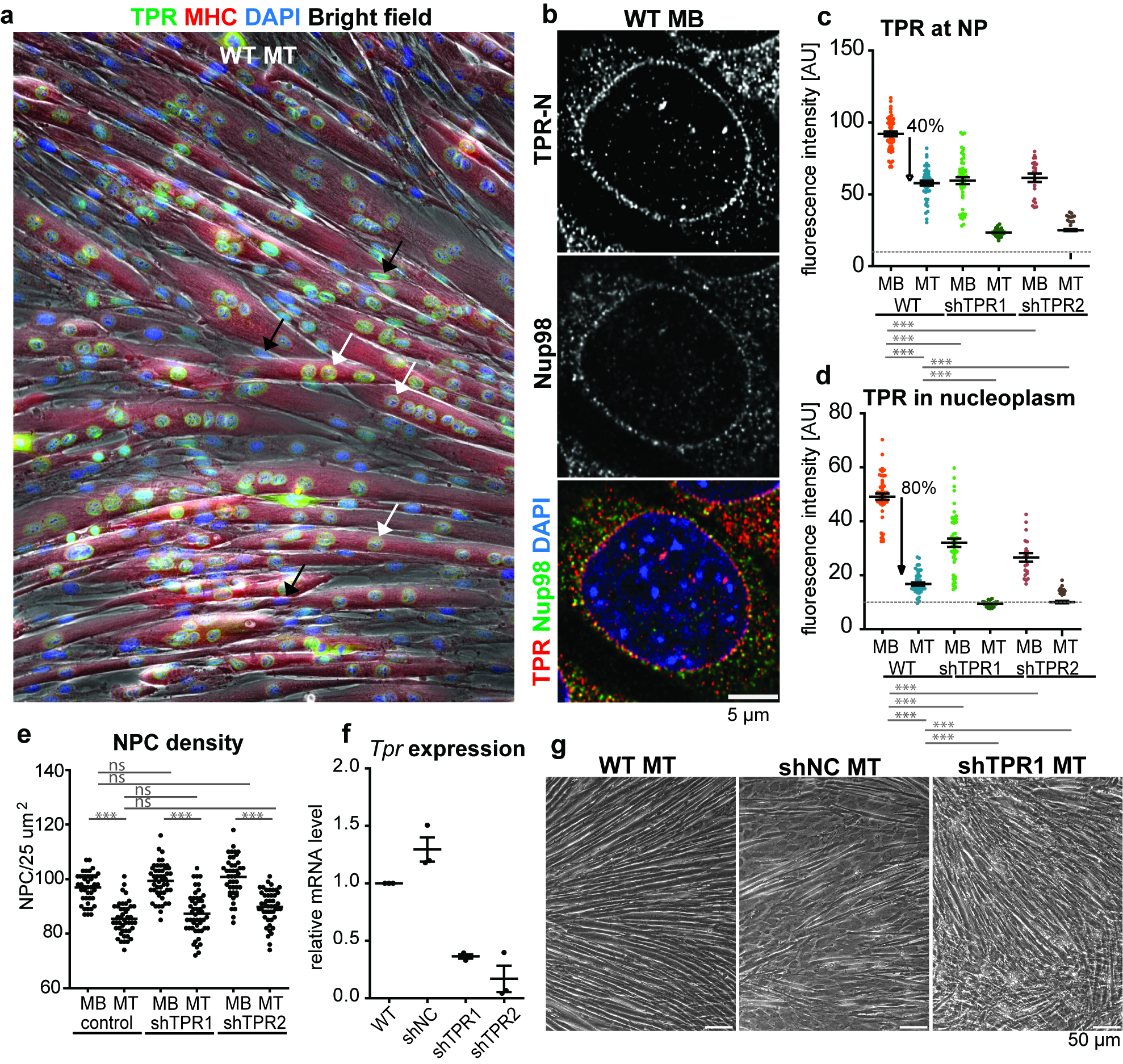

Supplement: Supplementary file 1 [file cells-10-01271-s001.zip › Supplemental/sFig1 - TPR localizes at NPCs and in the nucleoplasm in C2C12 as confirmed by the TPR-N antibody.tif]

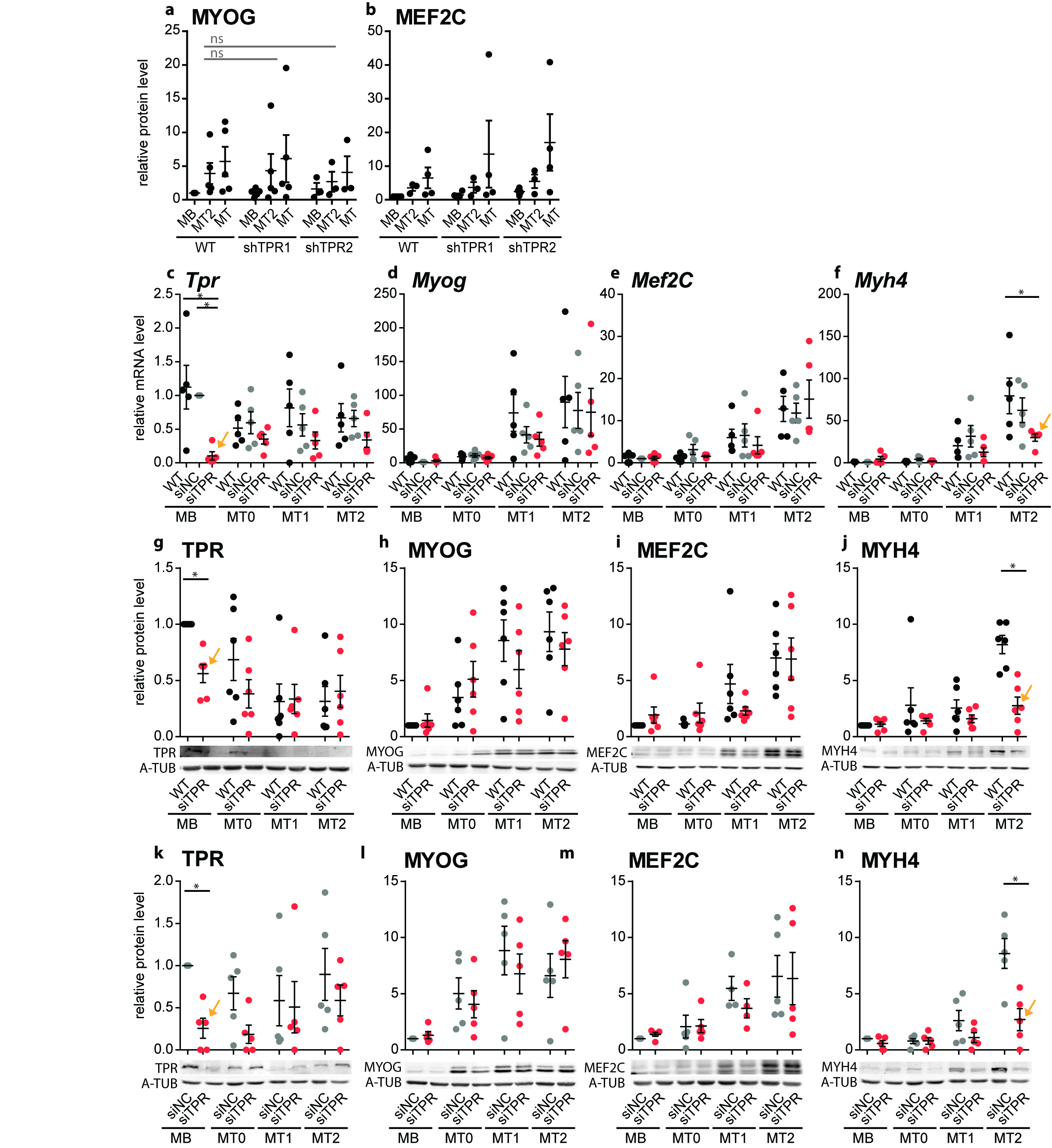

Supplement: Supplementary file 1 [file cells-10-01271-s001.zip › Supplemental/sFig2-MT2expression-siMyh4.tif]

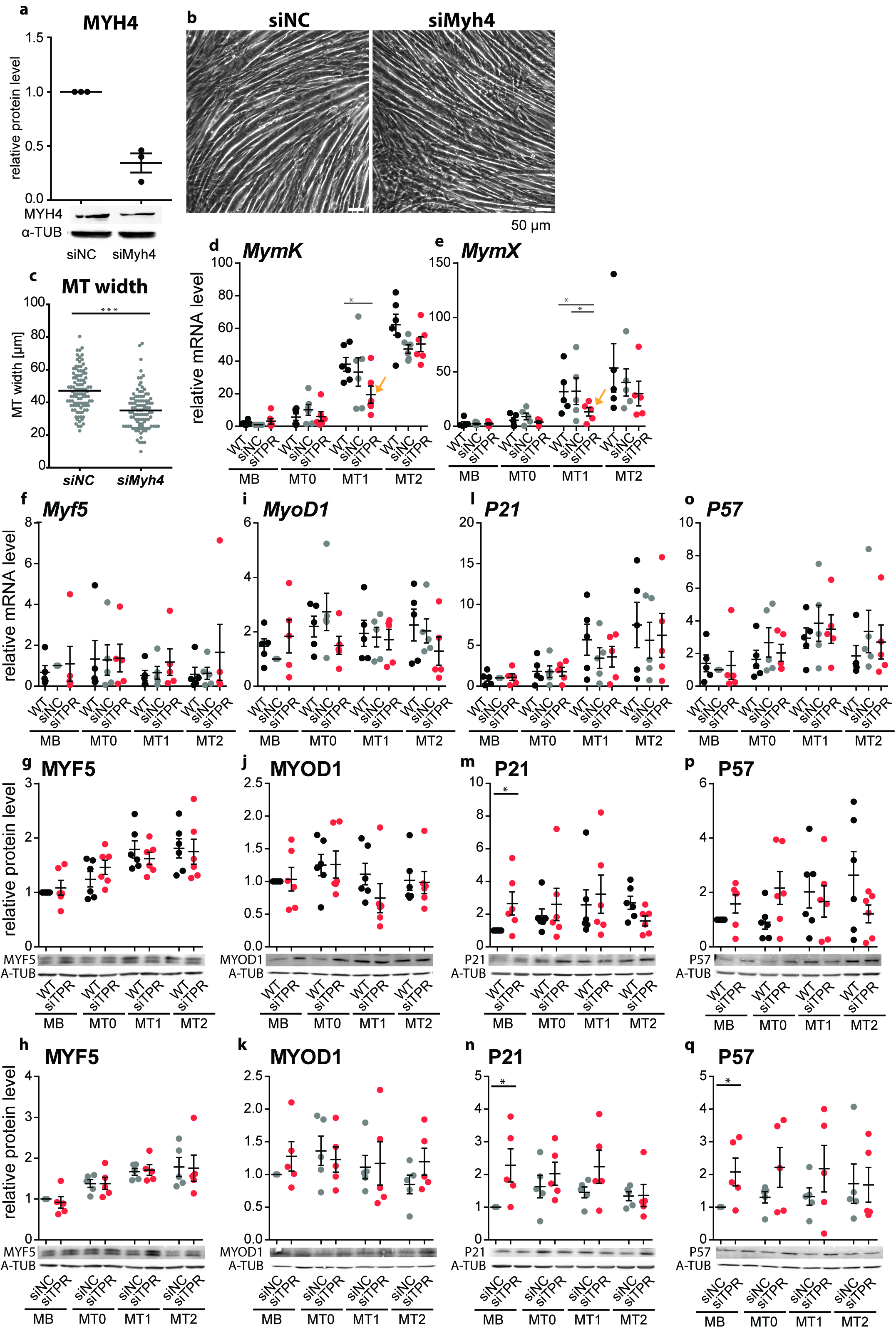

Supplement: Supplementary file 1 [file cells-10-01271-s001.zip › Supplemental/sFig3-siMyh4-Mymx-p57.tif]

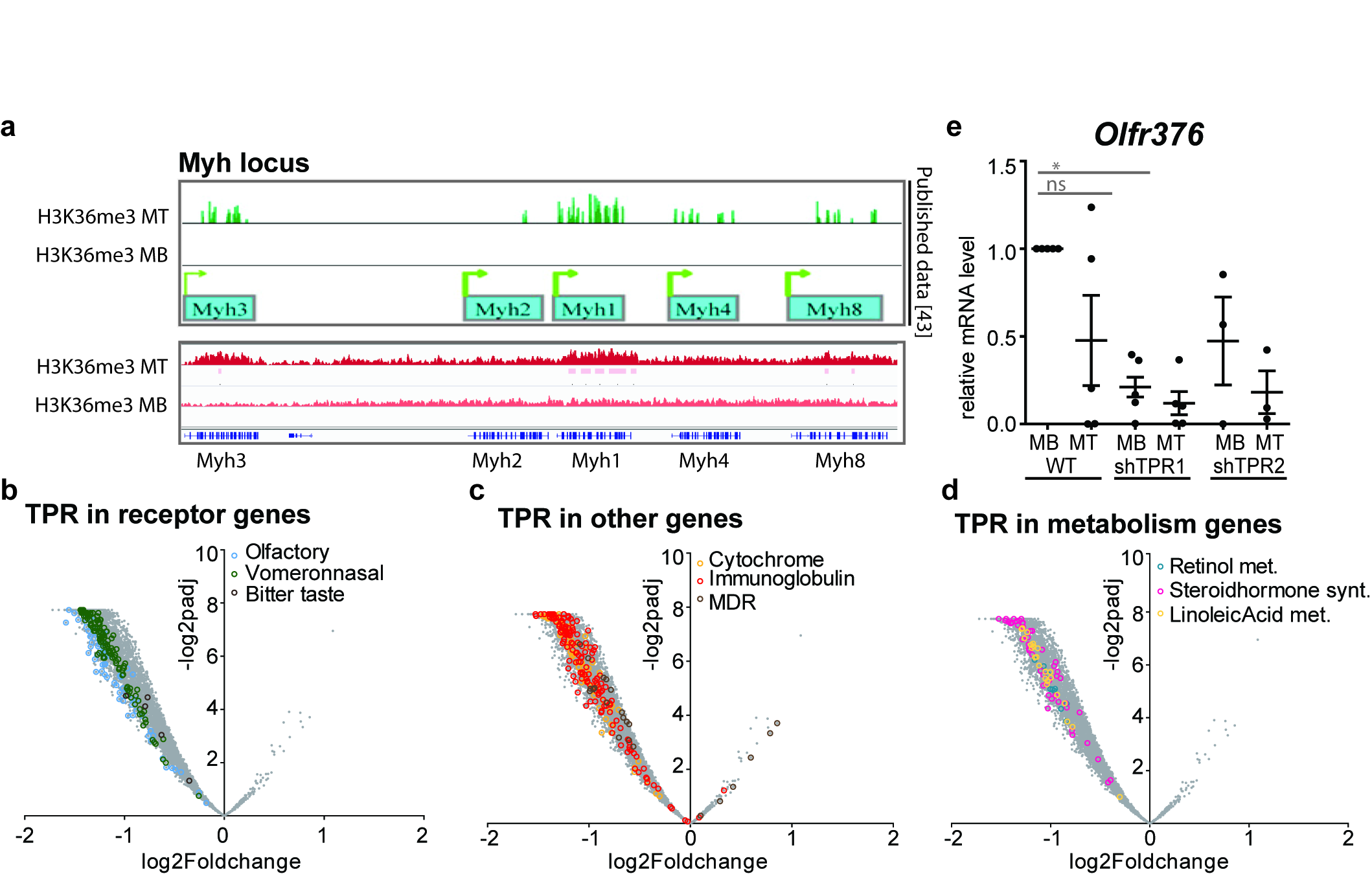

Supplement: Supplementary file 1 [file cells-10-01271-s001.zip › Supplemental/sFig4-ChIP-Seq analysis of TPR binding to the genome of C2C12 MBs and MTs.tif]

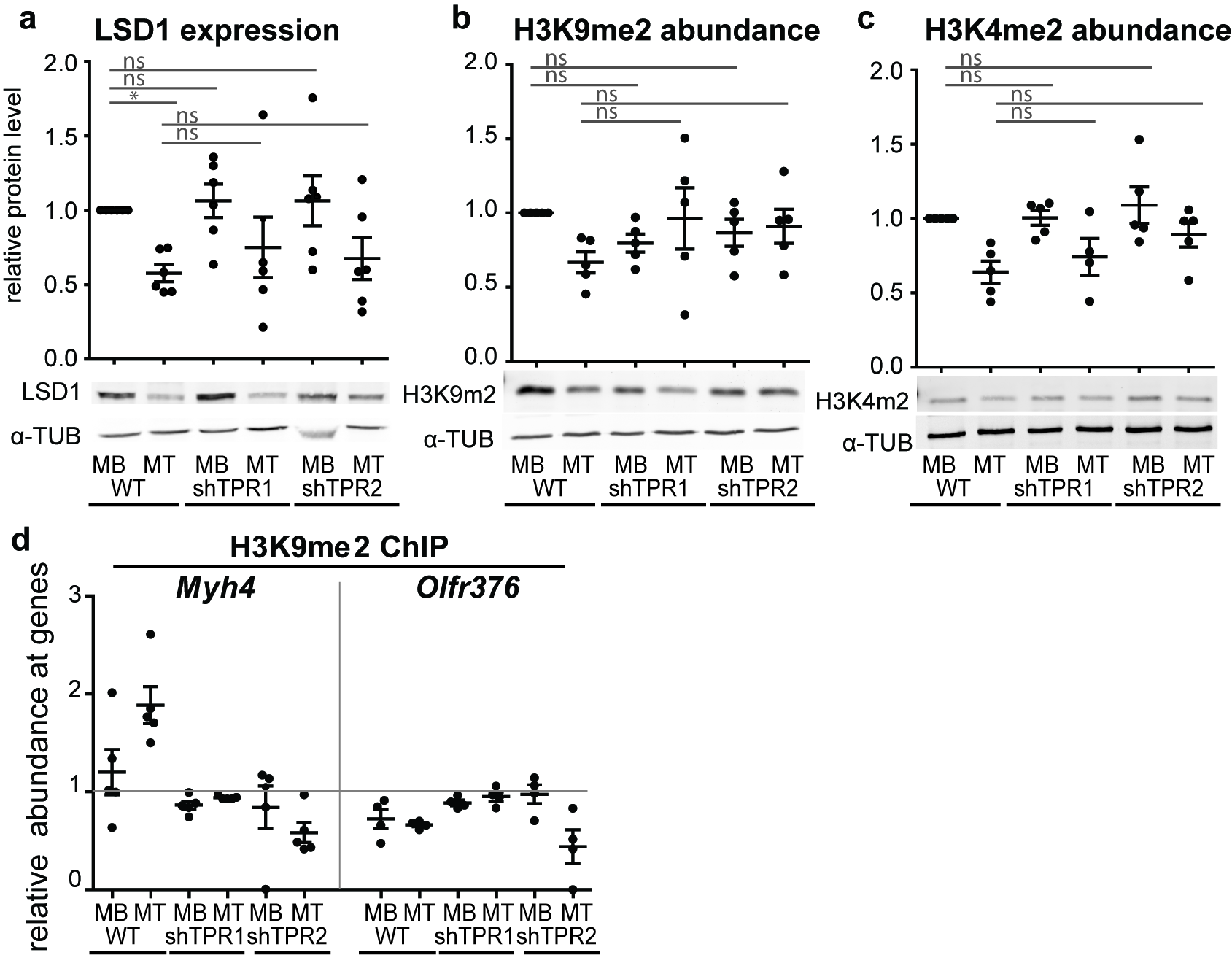

Supplement: Supplementary file 1 [file cells-10-01271-s001.zip › Supplemental/sFig5-TPR does not affect total protein levels of LSD1, H3K9me2 in C2C12 MBs and MTs.tif]
